# Picture quiz

**Published:** 2015

**Figure:**
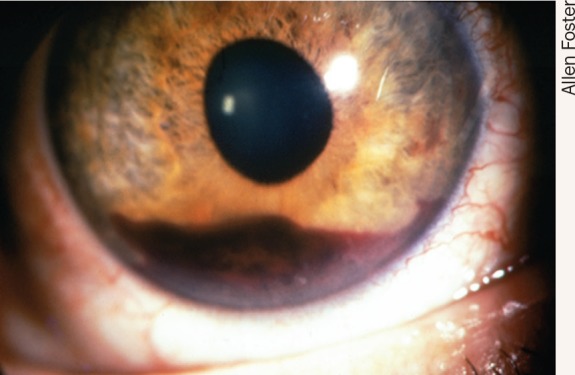


This is a picture of a 10-year-old boy who was hit in the eye by a stone.

**Q1.** What abnormality can you see on examination? (Select one)
  □ **a.** Corneal ulcer
  □ **b.** Hypopyon
  □ **c.** Orbital cellulitis
  □ **d.** Iritis
  □ **e.** Hyphaema
**Q2.** Which of these examinations/investigations would be appropriate? (Select all that apply)
  □ **a.** Ocular movements
  □ **b.** Examination of the pupils
  □ **c.** Slit lamp examination of the lens
  □ **d.** Measurement of intra-ocular pressure (IOP)
  □ **e.** Ophthalmoscopy
**Q3.** Which of the following may be indicated in treatment? (Select all that apply)
  □ **a.** Aspirin
  □ **b.** Immediate referral for surgical removal of the hyphaema
  □ **c.** Rest
  □ **d.** Acetazolamide tablets
  □ **e.** Annual check of intraocular pressure (IOP)

## ANSWERS

1. **e.** The picture shows blood in the anterior chamber, known as hyphaema, which can occur after blunt injuries.
2. **All of the tests are appropriate.** A blunt injury can cause a blow-out fracture of the orbit with entrapment of the inferior rectus muscle, causing diplopia and limitation of upward gaze. Blunt injuries can cause tears of the iris, whether iridodialysis or sphincter tears with traumatic mydriasis. The lens may be sub-luxated or dislocated by a blunt injury and there may be a concussion (traumatic) cataract. The IOP may be raised due to blood in the anterior chamber or damage to the trabecular meshwork (angle recession). Blunt injury can cause macula oedema or retinal dialysis.
3. **c, d and e.** Aspirin is contra-indicated as it may cause further bleeding. For pain anagement, paracetamol or ibuprofen are recommended. Unlike a penetrating injury, urgent surgical repair is not required, as most hyphaema will resolve without any further intervention. Rest is appropriate to allow the haemorrhage to resolve. If the IOP is raised then acetazolamide tablets may be indicated to reduce aqueous secretion. A hyphaema can cause damage to the trabecular meshwork, which increases the risk of glaucoma in the future. Annual IOP checks will detect glaucoma at an early stage, before it causes irreversible sight loss.

